# Association between *Methylenetetrahydrofolate Reductase C677T* Polymorphism and Susceptibility to Cervical Cancer: A Meta-Analysis

**DOI:** 10.1371/journal.pone.0055835

**Published:** 2013-02-19

**Authors:** Lili Yu, Kai Chang, Jian Han, Shaoli Deng, Ming Chen

**Affiliations:** 1 Department of Obstetrics and Gynecology, Institute of Surgery Research, Daping Hospital, The Third Military Medical University, Chongqing, China; 2 Department of Laboratory Medicine, Institute of Surgery Research, Daping Hospital, The Third Military Medical University, Chongqing, China; University of Georgia, United States of America

## Abstract

**Background:**

To assess the association between *MTHFR* polymorphism and cervical cancer risk, a meta-analysis was performed.

**Methods:**

Based on comprehensive searches of the PubMed, Embase, and Web of Science databases, we identified outcome data from all articles estimating the association between *MTHFR* polymorphism and cervical cancer risk. The pooled odds ratio (OR) with 95% confidence intervals (CIs) were calculated.

**Results:**

A total of 12 studies with 2,924 cases (331 cervical intraepithelial neoplasia (CIN) I, 742 CIN II/III, 1851 invasive cervical cancer) and 2,581 controls were identified. There was no significant association between *MTHFR* C677T polymorphism and CIN I risk (T vs. C, OR = 1.10, 95% CI = 0.92–1.31; TT vs. CC, OR = 1.14, 95% CI = 0.78–1.68; TT+CT vs. CC, OR = 1.22, 95% CI = 0.94–1.58; TT vs. CT+CC, OR = 0.99, 95% CI = 0.70–1.40). For the CIN II/III, lack of an association was also found (T vs. C, OR = 1.08, 95% CI = 0.95–1.23; TT vs. CC, OR = 1.15, 95% CI = 0.87–1.52; TT+CT vs. CC, OR = 1.13, 95% CI = 0.94–1.35; TT vs. CT+CC, OR = 1.07, 95% CI = 0.83–1.38). The T allele had significant association to susceptibility of invasive cervical cancer in recessive model (TT vs. CT+CC, OR = 1.23, 95% CI = 1.02–1.49). On subgroup analysis by ethnicity, similarly significant differences in T vs. C, TT vs. CC, and recessive model were found in Asians.

**Conclusion:**

The present meta-analysis suggested that *MTHFR* C677T polymorphism were to substantially contribute to invasive cervical cancer in recessive model.

## Introduction

Cervical cancer continues a serious threat to women throughout the world [1]. As the third most common cancer in women, it is estimated that there are nearly 530,232 new cases and 275,008 deaths die of cervical cancer in 2008 [2]. Epidemiological observations have established an aetiological association between human papillomavirus (HPV) infection and cervical cancer [3]–[4]. However, only a small percentage of infected women will ever develop cervical cancer. Therefore, infection with HPV alone is not sufficient for the development of cervical cancer and host genetic susceptibility, combined with lifestyle factors, may play a crucial role in exploring the progression of disease [5].

Methylenetetrahydrofolate reductase (MTHFR), a homodimeric enzyme, catalyzes the conversion of 5,10-methylenetetrahydrofolate to 5-methyltetrahyd- rofolate [6]. The enzyme plays a critical role in regulating the metabolism of folate and methionine, both being involved in DNA methylation and DNA synthesis required for normal development and growth [7]. The most common polymorphism, C-to-T transition at nucleotide 677 (C677T), is located on chromosome 1p36. This transition had been found to affect the catalytic domain of the MTHFR, thus reduce folate levels and elevate homocysteine levels [8]–[9]. Low folate levels may cause several cancers by influence DNA methylation and DNA synthesis [10]–[11]. Therefore, the *MTHFR* gene might be one of the candidate genes for susceptibility of cervical cancer.

A relatively large number of studies evaluated the association between *MTHFR* C677T polymorphism and cervical cancer risk. However, the *MTHFR C677T* polymorphism’s association with cervical cancer, or the lack thereof, remain inconclusive. To derive a more comprehensive and precise estimation of the relationship, we carried out a meta-analysis on all eligible case-control studies to estimate the effect of *MTHFR* polymorphism on the risk of cervical cancer.

## Results

### Study Characteristics

Twelve publications, including 2,924 cases (331 cervical intraepithelial neoplasia (CIN) I patients, 742 CIN II/III patients, 1851 invasive cervical cancer patients) and 2,581 controls, met the inclusion criteria [11]–[22]. A flowchart detailing the process for study identification and selection is shown in Fig. S1. The sample sizes ranged from 95 to 1546 patients (median 260.5, Interquartile range 161.5–777.5). Five of the 12 included studies evaluated the association between *MTHFR C677T* polymorphism and susceptibility of CIN I [12], [18], [20]–[22]. Six studies evaluated the association between *MTHFR C677T* polymorphism and susceptibility of CIN II/III [12], [14], [18], [20]–[22]. Eleven studies evaluated the association between *MTHFR C677T* polymorphism and susceptibility of cervical cancer [11]–[21]. The Newcastle-Ottawa Scale (NOS) scores ranged from 6 to 9, which indicated that the methodological quality was generally good. The genotype distribution in the controls of all studies was in agreement with Hardy-Weinberg equilibrium (HWE). The main characteristics of the studies were shown in Table 1.

**Table 1:** Association between individual study characteristics and *MTHFR C677T* polymorphism.

Study	Country	Ethnicity	Genetic type	Mean age, yearcases/controls	CIN I			CIN II/III			Invasive cancer			Control			Scores
					CC	CT	TT	CC	CT	TT	CC	CT	TT	CC	CT	TT	
Mostowska et al.	Poland	Caucasian	C677T	54.6/53.3							56	59	9	69	81	18	9
Prasad et al.	India	Mixed	C677T	NA/NA							57	5	0	116	8	1	6
Tong et al.	Kerea	Asian	C677T	50.8/45.7	52	82	25	54	74	32	53	65	28	152	198	77	8
Kohaar et al.	India	Caucasian	C677T	49.4/48.2				28	11	0	113	47	4	161	65	5	7
Nandan et al.	India	Mixed	C677T	NA/NA							36	0	26	53	0	24	8
Shekari et al.	India	Caucasian	C677T	48.6/48.8							125	68	7	170	28	2	7
Ma et al.	China	Asian	C677T	52.5/50.6							20	53	38	33	60	18	7
Kang et al.	Kerea	Asian	C677T	NA/NA							27	32	20	30	32	12	7
Zoodsma et al.	Netherlands	Caucasian	C677T	NA/NA	27	21	6	121	120	23	357	230	49	273	262	57	8
Sull et al.	Kerea	Asian	C677T	50.3/46.2	10	22	8	50	90	36	73	115	58	153	221	80	7
Lambropoulos et al.	Greece	Caucasian	C677T	33.2/33.2	20	28	5	27	29	8	11	8	2	42	37	12	6
Piyathilake et al.	USA	Mixed	C677T	30.4/23.9	6	13	6	11	23	5				16	12	3	7

Abbreviations and definitions: CIN, cervical intraepithelial neoplasia; *MTHFR, methylenetetrahydrofolate reductase*; NA, not available.

### The *MTHFR C677T* Polymorphism and CIN I Susceptibility

**Figure 1:**
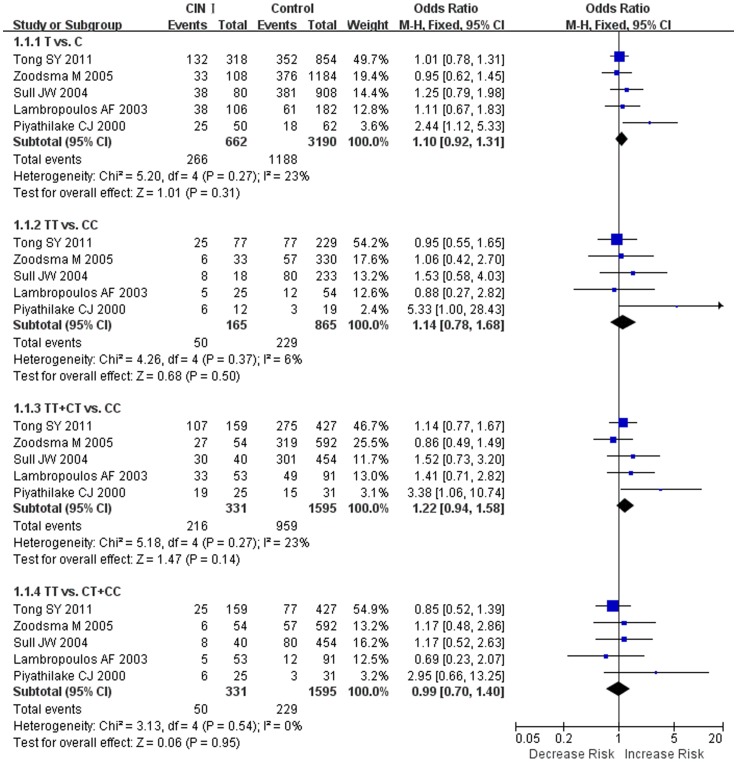
Forest plot of the overall risk of CIN I associated with the *MTHFR C677T* polymorphism. No significant association was found between the *MTHFR C677T* polymorphism and CIN I risk in all genetic models. A, T vs. C; B, TT vs. CC; C, dominant genetic model; D, recessive genetic model. Error bars indicate 95% CI. Solid squares represent each study in the meta-analysis. Solid diamonds represent pooled OR.

Fixed effects models were used to calculate the pooled OR in all genetic models. Overall, the combined results showed that no significant association was found in all genetic models (OR = 1.10, 95% CI = 0.92–1.31 for T vs. C, OR = 1.14, 95% CI = 0.78–1.68 for TT vs. CC, OR = 1.22, 95% CI = 0.94–1.58 for TT+CT vs. CC, and OR = 0.99, 95% CI = 0.70–1.40 for TT vs. CT+CC). Forest plots on the basis of all studies were shown in Fig. 1.

### The *MTHFR C677T* Polymorphism and CIN II/III Susceptibility

**Figure 2:**
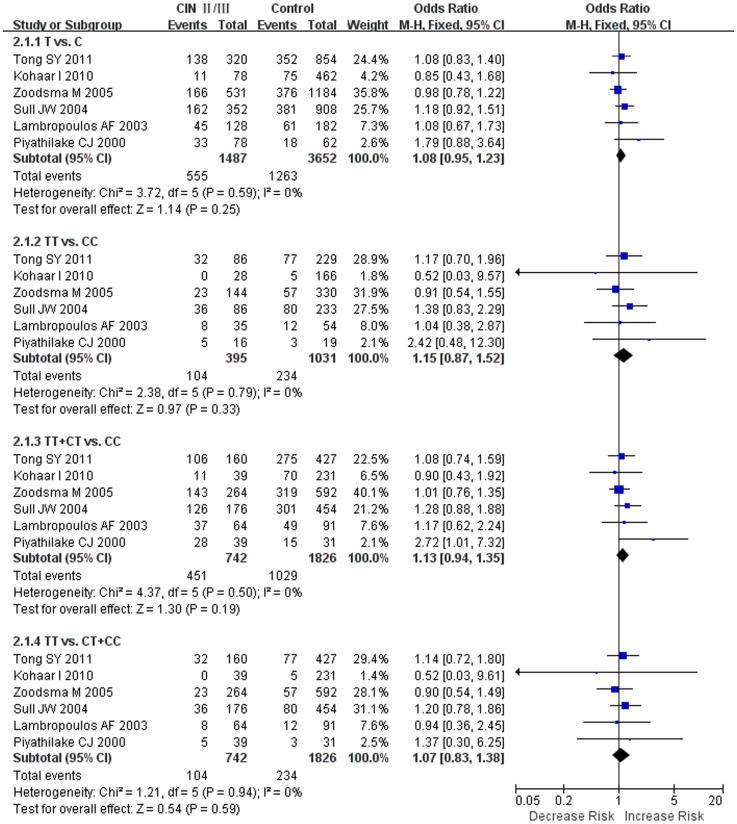
Forest plot of the overall risk of CIN II/III associated with the *MTHFR C677T* polymorphism. No significant association was found between the *MTHFR C677T* polymorphism and of CIN II/III risk in all genetic models. A, T vs. C; B, TT vs. CC; C, dominant genetic model; D, recessive genetic model. Error bars indicate 95% CI. Solid squares represent each study in the meta-analysis. Solid diamonds represent pooled OR.

The results on the *MTHFR C677T* polymorphism indicated that the T allele had no significant association to CIN II/III susceptibility as compared to the C allele under the fixed effects models (Fig. 2). The results were as followed: T vs. C (OR = 1.08, 95% CI = 0.95–1.23), TT vs. CC (OR = 1.15, 95% CI = 0.87–1.52), TT+CT vs. CC (OR = 1.13, 95% CI = 0.94–1.35), TT vs. CT+CC (OR = 1.07, 95% CI = 0.83–1.38).

### The *MTHFR C677T* Polymorphism and Invasive Cervical Cancer Susceptibility

**Figure 3:**
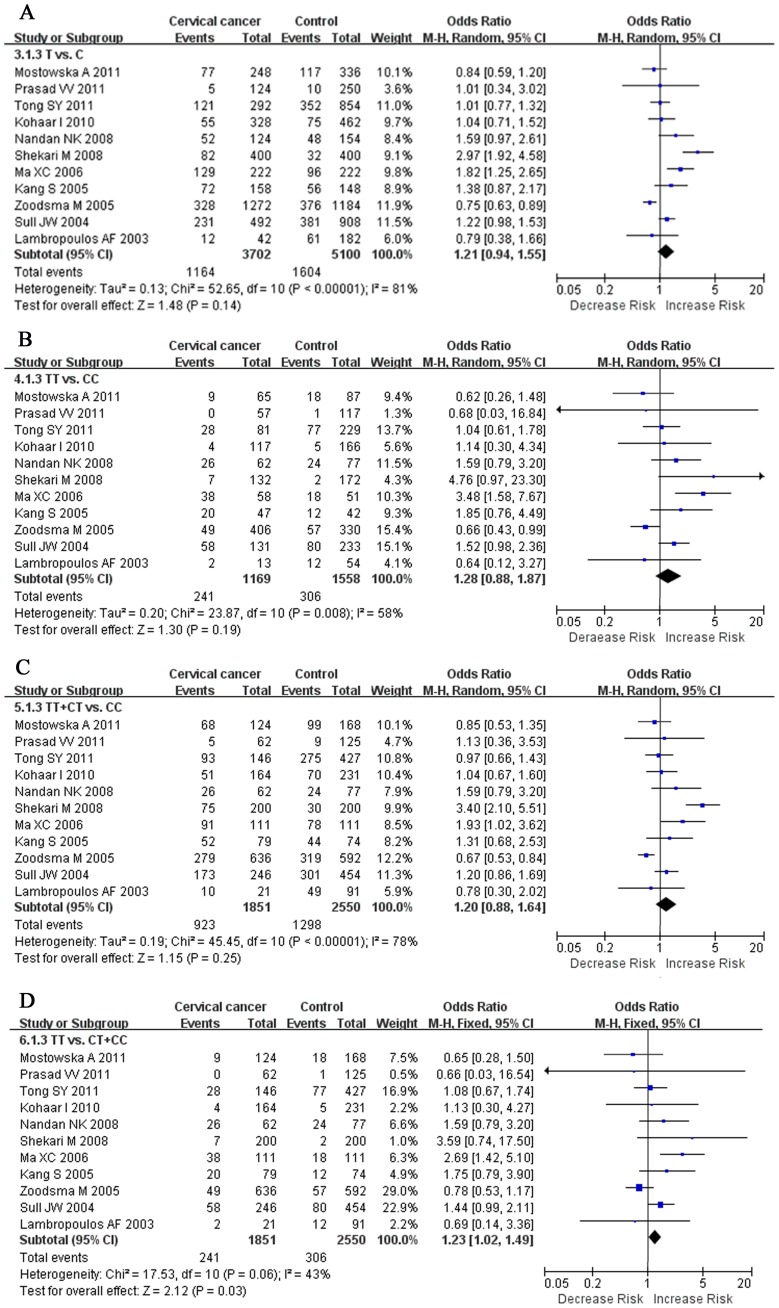
Forest plot of the overall risk of cervical cancer associated with the *MTHFR C677T* polymorphism. Significant association was found between the *MTHFR C677T* polymorphism and cervical cancer risk in recessive genetic model. A, T vs. C; B, TT vs. CC; C, dominant genetic model; D, recessive genetic model. Error bars indicate 95% CI. Solid squares represent each study in the meta-analysis. Solid diamonds represent pooled OR.

Fig. 3 showed that *MTHFR C677T* polymorphism was no significantly associated with invasive cervical cancer in T vs. C (OR = 1.21, 95%CI = 0.94–1.55), TT vs. CC, (OR = 1.28, 95% CI = 0.88–1.87), and TT+CT vs. CC (OR = 1.20, 95% CI = 0.88–1.64). The combined results showed significant differences in recessive model (TT vs. CT+CC, OR = 1.23, 95% CI = 1.02–1.49). When stratified by ethnicity, we observed a wide variation of T allele frequencies between the controls across different ethnicities. The result of One-way ANOVA indicated that the T allele frequencies were significant difference in Caucasians, Asians, and Mixed populations (P = 0.015). When meta-analysis was performed to assess association between *MTHFR C677T* polymorphism and different ethnicities, the T allele of *MTHFR C677T* polymorphism had significant association with invasive cervical cancer susceptibility in Asians. The results were showed in Table 2.

**Table 2:** Meta-analyses of *MTHFR C677T* polymorphism and risk of cervical cancer in each subgroup.

Category	T vs. C		TT vs. CC		Dominant model		Recessive model	
	OR(95%CI)	*I* ^2^(%)	OR(95%CI)	*I* ^2^ (%)	OR(95%CI)	*I* ^2^(%)	OR(95%CI)	*I* ^2^(%)
Ethnicity								
Caucasian	1.09(0.68–1.74)	88	0.76(0.54–1.06)	36	1.10(0.61–1.99)	89	0.84(0.61–1.17)	0
Asian	1.28(1.02–1.62)	53	1.66(1.05–2.62)	53	1.20(0.96–1.50)	11	1.51(1.17–1.94)	42
Mixed	1.48(0.94–2.31)	0	1.53(0.78–3.01)	0	1.45(0.80–2.62)	0	1.53(0.77–3.01)	0
SA	1.18(0.90–1.54)	85	1.19(0.79–1.81)	68	1.18(0.84–1.66)	82	1.16(0.96–1.41)	59

Abbreviations and definitions: CI, 95% confidence intervals; OR, odds ratio; SA: sensitivity analysis.

### Heterogeneity Analysis

For the association between *MTHFR C677T* polymorphism and invasive cervical cancer susceptibility, there were statistically significant heterogeneity in T vs. C (*I* ^2^ = 81%, *P*_Q_<0.00001), TT vs. CC (*I* ^2^ = 61%, *P*_Q_ = 0.005), dominant genetic model (*I* ^2^ = 78%, *P*_Q_<0.00001), and recessive genetic model (*I* ^2^ = 50%, *P*_Q_ = 0.03).

**Figure 4:**
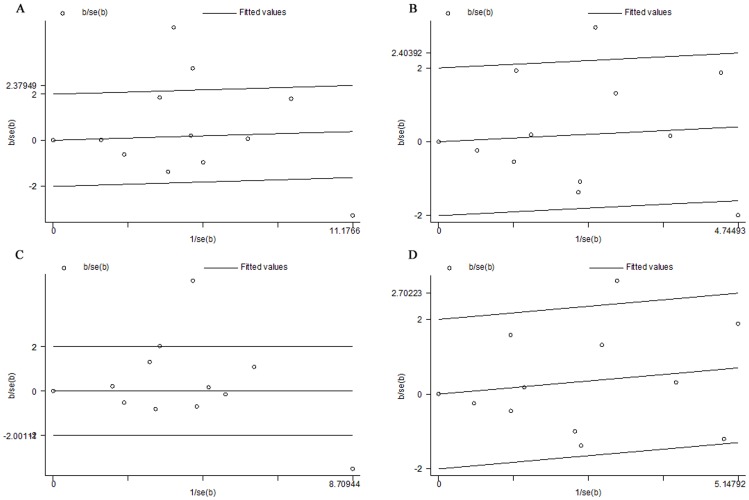
Galbraith plot of *MTHFR C677T* polymorphism and cervical cancer risk . A, The three studies of Shekari M et al., Ma XC et al., and Zoodsma M et al. were outliers in the T vs. C; B, The two studies of Ma XC et al. and Zoodsma M et al. were outliers in the TT vs. CC; C, The three studies of Shekari M et al., Ma XC et al., and Zoodsma M et al. were outliers in dominant genetic model; D, The study of Ma XC et al. was outliers in the recessive genetic model.

To explain the heterogeneity, Galbraith plots were performed in all genetic models. Galbraith plots [23] provide a graphical display to obtain a visual impression of the amount of heterogeneity from a meta-analysis. The position of each trial on the horizontal axis gives an indication of the weight allocated to it in a meta-analysis. The position on the vertical axis gives the contribution of each trial to the Q statistic for heterogeneity. In the absence of heterogeneity, we could expect all the points to lie within the confidence bounds (positioned 2 units over and below the regression line). In this meta-analysis, the three studies of Shekari M et al., Ma XC et al., and Zoodsma M et al. were outliers in the T vs. C and dominant genetic model (Fig. 4A, C). The two studies of Ma XC et al. and Zoodsma M et al. were outliers in the TT vs. CC (Fig. 4B). The study of Ma XC et al. was outliers in the recessive genetic model (Fig. 4D). When the studies of Shekari M et al., Ma XC et al., and Zoodsma M et al. were excluded respectively, all *I* ^2^ values were less than 50% and *P*_Q_ were greater than 0.1 (Table 3). The significant of pooled OR showed significant differences in TT vs. CC (OR = 1.31, 95% CI = 1.01–1.69).

**Table 3:** Meta-analyses of *MTHFR C677T* polymorphism and cervical cancer susceptibility after omitting the studies.

Polymorphism	OR (95% CI)	Z	P _OR_	*I* ^2^ (%)	*P*_Q_	Effect model
T vs. C^a^	1.11 (0.97, 1.26)	1.55	0.12	6	0.38	F
TT vs. CC^b^	1.31 (1.01, 1.69)	2.05	0.04	5	0.40	F
TT+CT vs. CC^a^	1.12 (0.95, 1.33)	1.34	0.18	0	0.56	F
TT vs. CT+CC^c^	1.13 (0.92, 1.38)	1.20	0.23	19	0.27	F

Abbreviations and definitions: CI, 95% confidence intervals; OR, odds ratio; *P*_Q,_ P value of Q test for heterogeneity; F, fixed-effect models.

a *MTHFR C677T* polymorphism and cervical cancer susceptibility after excluding the three studies of Shekari M et al., Ma XC et al., and Zoodsma M et al.

b *MTHFR C677T* polymorphism and cervical cancer susceptibility after excluding the two studies of Ma XC et al. and Zoodsma M et al.

c *MTHFR C677T* polymorphism and cervical cancer susceptibility after excluding the study of Ma XC et al.

### Sensitivity Analysis

Robustness of our results with regard to different assumptions was examined by performing a sensitivity analysis. Sensitivity analysis was performed based on the high NOS score (≥7). Two studies with relatively low NOS score (<7) were excluded from the sensitivity analysis. The sensitivity analysis indicated the results of our meta-analysis were relatively consistent even when some studies were excluded. The results were shown in Table 2.

### Publication Bias

Publication bias was estimated by the funnel plots. As shown in Fig. S2, the shape of the funnel plots revealed asymmetry in some degree due to the limited number of literatures. Then, Egger’s linear regression test was used to provide statistical evidence of funnel plots asymmetry. The result still did not suggest any evidence of publication bias.

## Discussion

Worldwide study has indicated that folate levels show a protective role in a variety of cancers. Owing to the importance of *MTHFR* in maintaining folate homeostasis, the *MTHFR C677T* polymorphism has been investigated in certain types of cancer, which included Colorectal, Thyroid, Breast, Ovarian, and cervical cancers [24]. The association between *MTHFR C677T* polymorphism and cervical cancer risk was first reported in a mixed populations by Piyathilake et al [22]; however, as discussed above, conflicting data regarding the role of *MTHFR* in cervical cancer susceptibility and presentation have been reported by series of case-control studies [11]–[14], [16], [18]–[21]. Against this backdrop, we performed a meta-analysis to clarify the relationship between *MTHFR C677T* polymorphism and cervical cancer risk.

In this meta-analysis, 12 studies (5 subgroups for CIN I, 6 subgroups for CIN II/III, and 11 subgroups for invasive cervical cancer) on *MTHFR C677T* polymorphism were performed to provide the most comprehensive assessment of the relationship between polymorphism and cervical cancer risk. The T allele of *MTHFR C677T* polymorphism had no association with the CIN I susceptibility for the T vs. C, TT vs. CC, dominant genetic model, and recessive genetic model in overall populations. Lack of an association was also found in CIN II/III and cervical cancer. In view of the complex effect of genetic polymorphisms on disease progression, the lack of an association between *MTHFR C677T* polymorphism and invasive cervical cancer susceptibility may attribute to other polymorphisms in *MTHFR* gene promoter which could affect the activity of MTHFR. Ulvik A et al. [25] demonstrated that *MTHFR A*1298*T* polymorphism was associated with reduced MTHFR activity. Meanwhile, the *MTHFR C677T* and *A*1298*T* polymorphisms appeared to interact with folate in determining cancer risk. Strong correlation between *MTHFR C677T* and *A*1298*T* polymorphisms was observed in cervical dysplasia as compared to normal cervical cytology [26]. In current study, we also performed meta-analysis to identify the association between *MTHFR A*1298*T* polymorphism and cervical cancer risk. There was no association between *MTHFR A*1298*T* polymorphism and cervical cancer risk (Table S1 and S2). Thus, the interaction between gene and gene might influence the association of *MTHFR* gene polymorphism with cervical cancer risk.

To explore a more precise relationship between *MTHFR C677T* polymorphism and invasive cervical cancer susceptibility, subgroup analysis by ethnicity was performed. First, we detected whether there was T allele frequency of variation in different ethnicities. The T allele frequency has significant differences in different populations. Next, the association between *MTHFR C677T* polymorphism and invasive cervical cancer risk in different ethnicities was explored. Lack of an association was also found in all genetic models.

In our meta-analysis, obvious heterogeneity was observed for the association between *MTHFR C677T* polymorphism and invasive cervical cancer risk. Then, we used the Galbraith plots to explore the sources of heterogeneity. We found all of the *I* ^2^ values were less than 50% and *P*_Q_ were greater than 0.1 after excluding the studies of Shekari M et al., Ma XC et al., and Zoodsma M et al. respectively. The results indicated that the three studies might be the major source of the heterogeneity for the association between *MTHFR C677T* polymorphism and cervical cancer risk. The results of subgroup analysis revealed that the ethnicity might contribute to the potential heterogeneity.

There are some limitations to this meta-analysis. Firstly, the retrieved literature is potentially not comprehensive enough. Studies included in our meta-analysis were limited to published articles. We did not track the unpublished articles to obtain data for analysis. Secondly, as many other factors such as age, parity, smoking, and alcohol consumption may participate in the progression of disease, we did not carry out subgroup analysis based on these factors due to limited data. Thirdly, the small sample sizes in some subgroup analyses limited the ability to draw more solid conclusions.

Conclusively, *MTHFR C677T* polymorphism may associate with genetic susceptibility of invasive cervical cancer in recessive model based on the current published studies. Similarly significant differences in T vs. C, TT vs. CC, and recessive model were found in Asians. Moreover, further studies with large sample size of different ethnic populations will be necessary to combine genetic factors together with age, parity, smoking, and alcohol consumption.

## Materials and Methods

### Data Sources and Search Strategy

This meta-analysis followed the Preferred Reporting Items for Systematic Reviews and Meta-analysis (PRISMA) criteria [27]. Two investigators (L.Y. and K.C.) independently performed a systematic electronic search of the PubMed, Embase, Web of Science databases for original articles published until 1 April, 2012 to identify potentially relevant articles and abstracts. Search terms used were “Methylenetetrahydrofolate reductase or MTHFR” and “cervical cancer or cervical carcinoma or uterine cervix cancer or cervical neoplasia or cervical dysplasia” and “polymorphism or mutation or variant”. There were no language restrictions. We reviewed the bibliographies of all selection articles to identify additional relevant studies.

### Selection of Publications

Two reviewers independently screened titles and abstracts of all studies for relevancy. Disagreements were resolved by a third opinion. Full-text publications were retrieved for relevant articles. The strength of the individual studies was weighed for relevance, based on the following items: (1) evaluation of the *MTHFR C677T* polymorphism and cervical cancer or its precursor lesion, CIN, (2) case-control studied, (2) sufficient data for estimating an odds ratio (OR) with 95% confidence intervals (CIs), (3) genotype distribution of control population in HWE, and (4) studies written in English or Chinese. For the studies with the same or overlapping data by the same authors, the most recent or largest population was selected.

### Data Extraction

Data were extracted independently from each study by two reviewers according to the inclusion criteria listed above. Agreement was reached after discussion for conflicting data. The following data were collected from each study: first author’s name, publication year, original country, ethnicity, control source, sample size, genotyping method, and genotype number in cases and controls.

### Quality Assessment

The quality of included studies was assessed independently by the same two investigators using the NOS [28]. The NOS uses a ‘star’ rating system to judge quality based on 3 aspects of the study: selection of study groups, comparability of study groups and ascertainment of the exposure of interest. Studies with a score of 7 stars or greater were considered to be of high quality.

### Statistical Analysis

The strength of association between *MTHFR* polymorphism and susceptibility of cervical cancer or CIN was estimated by OR and corresponding 95% CIs. The pooled OR was calculated respectively for T vs. C, TT vs. CC, dominant genetic model (TT+CT vs. CC), and recessive genetic model (TT vs. CT+CC). Between-study heterogeneity was assessed by the Q-test and *I* ^2^ test, *P*_Q_ <0.10 and *I* ^2^>50% indicated evidence of heterogeneity. Then, the random-effects model (the DerSimonian and Laird method)[29]–[30] was used to calculate the pooled OR. Otherwise, the fixed-effects model (Mantel-Haenszel) was adopted [31]. The forest plots were inspected to indicate the overall results, which show information from the individual studies that were included in the meta-analysis, and an estimate of the overall results. It also allows a visual assessment of the amount of variation between the results of the studies (heterogeneity).

Subgroup analyses were performed by ethnicity of study population. Sensitivity analysis was performed based on the high quality studies (according to the NOS score). Asymmetry funnel plots were inspected to assess potential publication bias. The Egger’s linear regression test was also used to assess publication bias statistically [32].

Data were analyzed by using STATA 11.0 (Stata Corporation, College Station, TX, USA) and Revman 5.0 (The Cochrane Collaboration).

## Supporting Information

Figure S1 **Flow diagram of the selection of eligible studies.**
(TIF)

Figure S2 **Funnel plots of all genetic models in overall studies.** A. T vs. C; B. TT vs. CC; C. dominant model (TT+CT vs. CC); D. recessive model (TT vs. CT+CC). Funnel plots of dominant model seemed asymmetry. Each point represents a separate study for the indicated association.
(TIF)

Table S1 **Association between individual study characteristics and** ***MTHFR A1298T*** **polymorphism.**
(DOC)

Table S2 **Meta-analyses of** ***MTHFR A1298T*** **polymorphism and risk of cervical cancer.**
(DOC)

PRISMA S1 **Checklist Association between methylenetetrahydrofolate reductase C677T polymorphism and susceptibility of cervical cancer.**
(DOC)
