# Supplementary figures and images for: Association between Methylenetetrahydrofolate Reductase C677T Polymorphism and Susceptibility to Cervical Cancer: A Meta-Analysis
Source: PLoS One. 2013 Feb 19;8(2):e55835. doi: 10.1371/journal.pone.0055835 (PMC3576378; doi:10.1371/journal.pone.0055835)

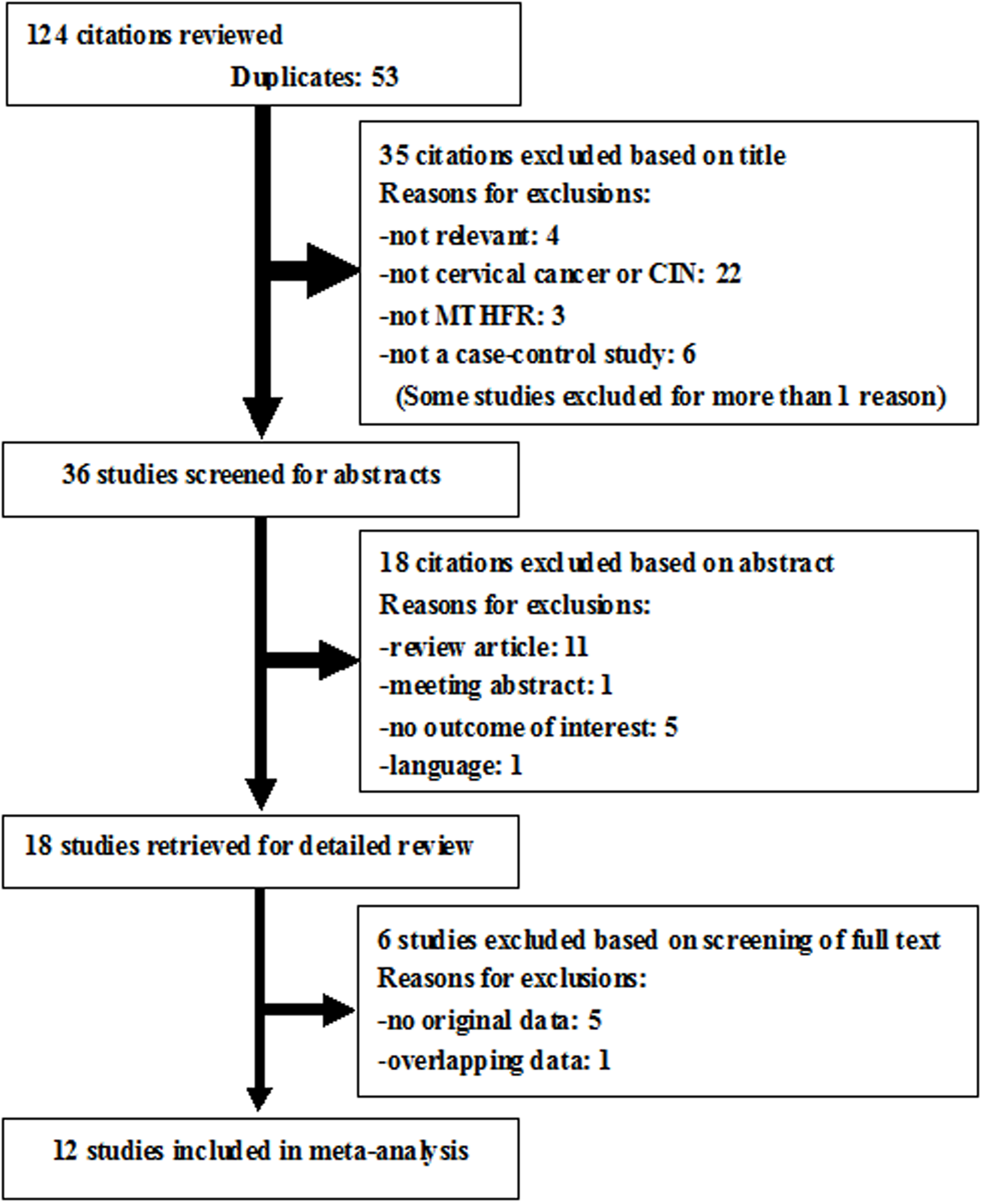

Supplement: Figure S1 — Flow diagram of the selection of eligible studies. (TIF) [file pone.0055835.s001.tif]

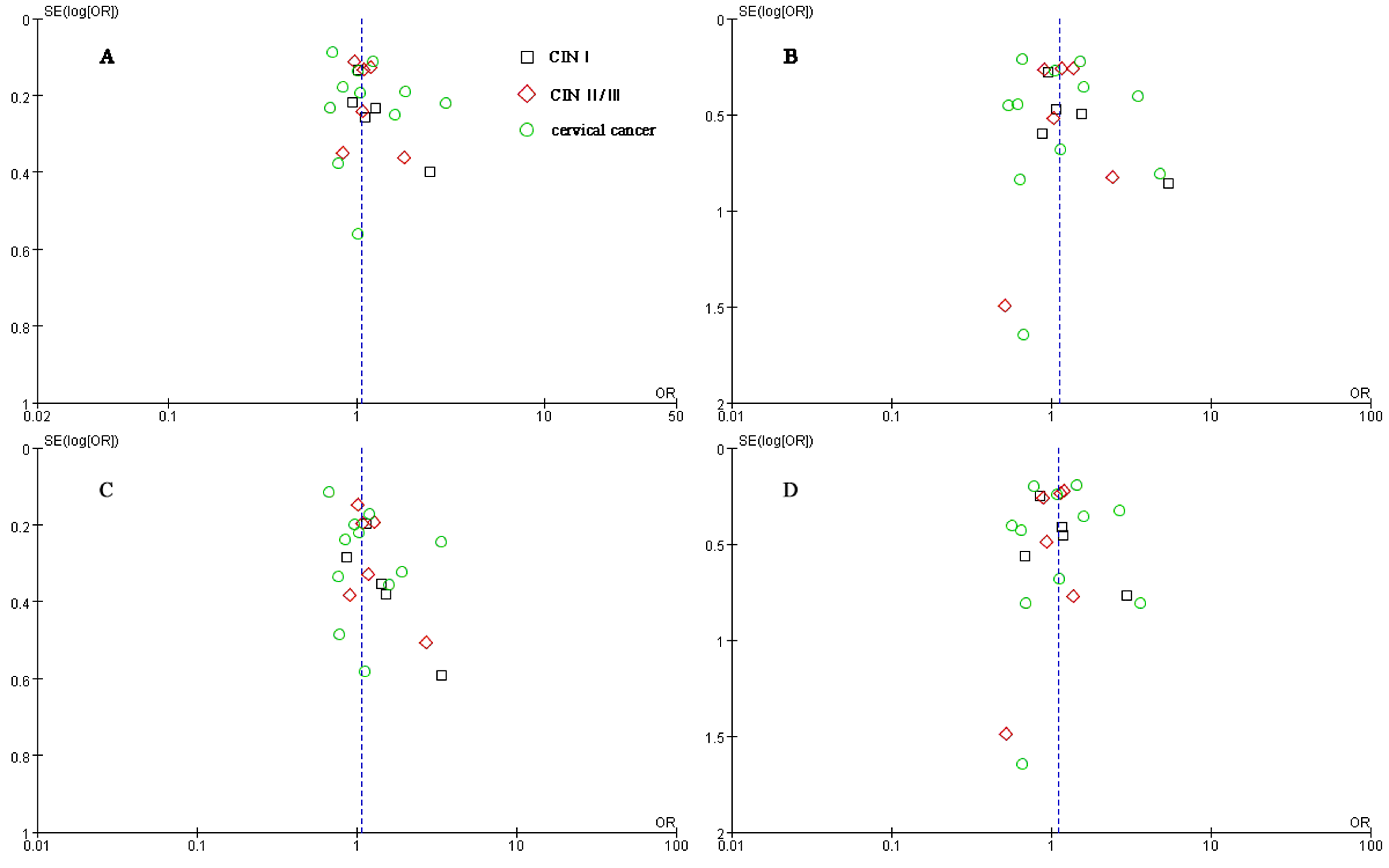

Supplement: Figure S2 — Funnel plots of all genetic models in overall studies. A. T vs. C; B. TT vs. CC; C. dominant model (TT+CT vs. CC); D. recessive model (TT vs. CT+CC). Funnel plots of dominant model seemed asymmetry. Each point represents a separate study for the indicated association. (TIF) [file pone.0055835.s002.tif]
