# Supplementary material for: Association between Methylenetetrahydrofolate Reductase C677T Polymorphism and Susceptibility to Cervical Cancer: A Meta-Analysis
Source: PLoS One. 2013 Feb 19;8(2):e55835. doi: 10.1371/journal.pone.0055835 (PMC3576378; doi:10.1371/journal.pone.0055835)
Supplement: Table S1 — Association between individual study characteristics and MTHFR A1298T polymorphism. (DOC) [file pone.0055835.s003.doc]

| Study | Country | Ethnicity | Genetic type | Mean age, year  cases/controls | CIN Ⅰ | | |  | CIN Ⅱ/Ⅲ | | |  | Invasive cancer | | |  | Control | | | Scores |
| --- | --- | --- | --- | --- | --- | --- | --- | --- | --- | --- | --- | --- | --- | --- | --- | --- | --- | --- | --- | --- |
|  |  |  |  |  | AA | AC | CC |  | AA | AC | CC |  | AA | AC | CC |  | AA | AC | CC |  |
| Tong et al. | Kerea | Asian | A1298C | 50.8/45.7 | 107 | 46 | 7 |  | 117 | 39 | 4 |  | 89 | 57 | 2 |  | 278 | 132 | 18 | 8 |
| Kohaar et al. | India | Caucasian | A1298C | 49.4/48.2 |  |  |  |  | 15 | 20 | 4 |  | 58 | 83 | 23 |  | 85 | 119 | 27 | 7 |
| Kang et al. | Kerea | Asian | A1298C | NA/NA |  |  |  |  |  |  |  |  | 55 | 22 | 2 |  | 58 | 25 | 1 | 7 |

**Table S1** Association between individual study characteristics and *MTHFR A1298T* polymorphism

Abbreviations and definitions: CIN, cervical intraepithelial neoplasia; *MTHFR, methylenetetrahydrofolate reductase*; NA, not available
