# Supplementary material for: Association between Methylenetetrahydrofolate Reductase C677T Polymorphism and Susceptibility to Cervical Cancer: A Meta-Analysis
Source: PLoS One. 2013 Feb 19;8(2):e55835. doi: 10.1371/journal.pone.0055835 (PMC3576378; doi:10.1371/journal.pone.0055835)
Supplement: Table S2 — Meta-analyses of MTHFR A1298T polymorphism and risk of cervical cancer. (DOC) [file pone.0055835.s004.doc]

| Category | Study number | T vs. C |  | TT vs. CC |  | Dominant model |  | Recessive model |
| --- | --- | --- | --- | --- | --- | --- | --- | --- |
|  |  | OR (95%CI) *I*^2^ (%) |  | OR (95%CI) *I*^2^ (%) |  | OR (95%CI) *I*^2^ (%) |  | OR (95%CI)  *I*^2^ (%) |
| CINⅠ | 1 | 0.95(0.68-1.31) NA |  | 1.01(0.41-2.49) NA |  | 0.92(0.62-1.35) NA |  | 1.04(0.43-2.54) NA |
| CINⅡ/Ⅲ | 2 | 0.77(0.58-1.03) 0 |  | 0.65(0.29-1.44) 0 |  | 0.73(0.52-1.04) 0 |  | 0.70(0.32-1.53) 0 |
| Invasive cancer | 3 | 1.07(0.87-1.31) 0 |  | 0.99(0.57-1.71) 28 |  | 1.12(0.86-1.45) 0 |  | 0.98(0.58-1.63) 39 |

**Table 2** Meta-analyses of *MTHFR A1298T* polymorphism and risk of cervical cancer

Abbreviations and definitions: CIN, cervical intraepithelial neoplasia; *MTHFR, methylenetetrahydrofolate reductase*; NA, not available
